# Emotion Regulation and Mental Health in Young Elite Athletes

**DOI:** 10.3390/sports13090284

**Published:** 2025-08-22

**Authors:** Fredrik Fröjdö Regborn, Stefan Holmström, Michael Svensson, Magnus Sjögren

**Affiliations:** 1 Department of Clinical Sciences, Umeå University, 90185 Umeå, Sweden; magnus.sjogren@umu.se; 2 Department of Psychology, Umeå University, 90187 Umeå, Sweden; stefan.holmstrom@umu.se; 3 Department of Community Medicine and Rehabilitation, Umeå University, 90187 Umeå, Sweden; michael.svensson@umu.se

**Keywords:** emotion regulation, mental health, athletes, expressive suppression, cognitive reappraisal

## Abstract

Emotion regulation strategies, specifically expressive suppression (ES) and cognitive reappraisal (CR), are known to influence mental health outcomes in the general population and adult elite athletes. Young elite athletes, who face unique academic and athletic pressures, remain understudied in this regard. The main aim of this study was to examine the relationship between ES and CR and mental health in young elite athletes. This longitudinal study included 93 young elite athletes (aged 15–17) attending upper secondary education in Sweden. Participants completed the Emotion Regulation Questionnaire and the General Health Questionnaire at three time points over 12 months. Linear mixed models were used to examine associations between ES, CR, and mental health. Higher use of ES was significantly associated with poorer mental health (*β* = 0.34, *p* < 0.001), while greater use of CR predicted better mental health (*β* = −0.33, *p* < 0.001) across the study period. Expressive suppression and CR are both important for the mental health of young elite athletes, with CR being protective and ES conferring risk. Given the observed associations, future research could examine whether interventions aiming to enhance CR and reduce ES are linked to better mental health in young elite athletes.

## 1. Introduction

Mental health problems are a prevalent issue among elite athletes, with up to 57 percent experiencing symptoms of anxiety or depression [1,2]. The repercussions of mental health problems in elite athletes are far-reaching, impacting various aspects of their lives, from decreased performance outcomes [3] to risk of suicide [4,5]. For young elite athletes, the problem may be even more pronounced, as research shows psychiatric disorders are more prevalent among elite athletes under 18 compared with older groups [6]. Stigma around seeking help is an additional barrier for young athletes [7], potentially delaying early intervention and reducing treatment engagement. This is especially concerning since adolescence is a critical period for the onset of many psychiatric disorders [8]. Given the unique pressures faced by this group [9], there is a pressing need for successful coping mechanisms. Emotion regulation, although less studied in young elite athletes, is one such strategy that may help buffer against the negative impact of these demands.

### 1.1. Emotion Regulation and Mental Health

Emotion regulation refers to the effort to influence what emotion we are feeling, when we feel it, and how the emotion is experienced and expressed [10]. The Emotion Regulation Questionnaire (ERQ) is widely used to assess two core strategies: cognitive reappraisal (CR), which involves reframing a situation to alter its emotional impact, and expressive suppression (ES), which entails inhibiting outward emotional expressions. CR is generally considered adaptive and associated with psychological well-being, while ES is viewed as maladaptive and linked to poorer mental health outcomes. Previous research confirms that greater use of CR has been associated with better mental health, whereas higher ES is associated with increased distress and reduced well-being in both general and athletic populations [11,12]. However, to the best of our knowledge, there is no previous research on these associations in young elite athletes participating in upper secondary education.

### 1.2. Changes in Emotion Regulation Strategies

Findings on developmental trajectories of emotion regulation strategies in adolescents are mixed. Some studies report a decline in maladaptive strategies such as suppression before age 16 [13,14], while others observe increases [15,16] or stability [13,17] in both CR and ES throughout adolescence [17,18]. These inconsistencies suggest that environmental factors—such as increased academic and athletic demands—may drive shifts in emotion regulation during this transition, rather than maturation alone. Notably, the first year of upper secondary education is marked by heightened stress and transition demands for young elite athletes [19], underscoring the importance of understanding how emotion regulation strategies evolve and impact mental health during this critical period.

### 1.3. The Role of the Coach and the Peers for Emotion Regulation Strategies

The interpersonal environment created by coaches and peers may play a significant role in shaping emotion regulation strategies among young elite athletes. Coaches influence athletes through their interpersonal style, which can either support or undermine basic psychological needs such as autonomy, structure, and involvement [20,21]. In the context of emotion regulation, previous research has indicated that the perceived support of these basic psychological needs is related to emotion regulation in athletes [22]. This means that need-supportive coaching styles, characterized by encouragement, clear communication, and respect, are likely linked to more adaptive emotion regulation, while controlling or critical approaches may promote maladaptive strategies like ES.

Peers also influence each other meaningfully by establishing the motivational climate within teams. A task-involving peer climate, where effort, cooperation, and personal improvement are valued, is associated with positive emotional and motivational outcomes. In contrast, an ego-involving climate—focused on outperforming others and penalizing mistakes [23,24]—can undermine motivation and well-being [25,26]. Given the strong influence of peer relationships during adolescence [27], understanding and fostering supportive peer climates is likely crucial for supporting adaptive emotion regulation and overall mental health in young elite athletes.

### 1.4. Study Aims and Hypotheses

The data for this study stems from the Dragon project, a longitudinal study of young elite athletes attending upper secondary education in Sweden. The aim of the present study was to examine within-individual changes in emotion regulation strategies and their association with mental health in young elite athletes over a 12-month period, while also considering the influence of coach interpersonal style and peer motivational climate.

Based on the literature, we formulated three hypotheses:

**H1.**  *ES will be related to worse mental health, while CR will be related to better mental health.*

**H2.**  *Overall use of emotion regulation strategies will increase during this 12-month period.*

**H3.**  *A need-supportive coach interpersonal style and task-involving peer motivational climate will be related to less ES and more CR, and a controlling coach interpersonal style and ego-involving peer motivational climate will be related to more ES and less CR*.

## 2. Materials and Methods

### 2.1. Participants

During the project, all students entering the sports high school Dragonskolan in 2013, 2014, or 2015 were invited to participate. No exclusion criteria were used. Almost all invited students agreed to participate. Final data from the cohort were collected in 2018. Measures were collected from questionnaires, DXA scans, blood samples, and medical records. The questionnaires were filled out twice per year. The data used in the present study were from the questionnaires from the first three time points of data collection, gathered from the autumn of 2013, their first semester, until the autumn of 2014, their third semester. The participants for this study consisted of 94 young elite student athletes, all part of the first wave of the Dragon project. One participant was omitted for leaving odd responses, leaving 93 participants (age range = 15–17 years; 56 [60.2%] males, 37 [39.8%] females). Main elite sport activities included both team sports (e.g., floorball, soccer, and ice hockey; 78 participants) and individual sports (e.g., athletics, tennis, and golf; 14 participants). One participant had missing data on the type of sport. Since the sample was predominantly composed of team sport athletes, this should be considered when interpreting the generalizability of findings to individual sport athletes. Participants trained on average 12.23 h per week (SD = 3.12; one outlier removed due to a reported training dose of 40 h per week) at baseline. This cohort of participants was selected due to having measures of emotion regulation on more than one occasion.

### 2.2. Power Analysis

Previous studies with adolescent samples have reported a correlation between ES and depressive symptoms of between *r* = 0.21 and *r* = 0.36, depending on gender and time point [28], as well as a correlation between CR and depressive symptoms of *r* = −20 [29]. According to a simulation study on power analysis for linear mixed models, with three measurement points and a medium intraclass correlation, a sample size of 30–80 participants is sufficient to detect such effect sizes, with a power of 0.80 and alpha of 0.05 [30]. Based on guidelines from Gignac and Szodorai [31] and data from the simulation study [30] sufficient sample sizes for minimal detectable effect sizes, with a power of 0.80, alpha of 0.05, a medium intraclass correlation, and three measurement points, would be *n >* 200 for a small effect size, *n* = 90 for a medium effect size, and *n* = 40 for a large effect size. In the present study, 93 participants contributed some data; however, the data contained some missing values due to either missing individual items or not being present at data collection. With 70 participants, effect sizes at *r* = 0.22 or larger could be detected. Since this sample consisted of an enriched population due to all being young elite athletes and likely being similar in terms of athletic performance pressure or motivation, effect sizes larger than *r* = 0.22 were expected.

### 2.3. Questionnaires

#### 2.3.1. Emotion Regulation Strategies

To measure emotion regulation strategies, the Emotion Regulation Questionnaire (ERQ) [32] was used. ERQ consists of a total of 10 items. Cognitive reappraisal is measured with six items, for example, “When I want to feel less negative emotion (such as sadness or anger), I change what I’m thinking about”, and expressive suppression is measured with four items, for example, “I control my emotions by not expressing them”. The regular version of ERQ was used, in which each item was rated on a Likert scale from 1 (strongly disagree) to 7 (strongly agree). Mean scores for each subscale were calculated. High scores indicate more use of that strategy. Since all participants were over 15 years old, and ERQ-CA has not been validated in Swedish, the adult version of the ERQ was used. The Swedish version has shown Cronbach’s alpha of 0.81 and 0.73 for CR and emotion regulation, respectively. as well as an acceptable fit for the two-factor model [33]. In the present study, Cronbach’s alpha was measured at 0.76, 0.87, and 0.88 for CR and 0.44, 0.68, and 0.73 for ES at T1, T2, and T3, respectively. The low internal consistency of ES at T1 represents a significant limitation that may affect the validity of findings at this time point. This should be considered when interpreting results, particularly those involving T1 ES scores. The improved consistency at later time points suggests measurement stability improved over time.

Due to this low internal consistency of ES at T1, further analyses of internal consistency were conducted to diagnose the errors (see Appendix B for detailed analysis). Reanalysis was conducted without item 9 at T1. There were some differences in the coefficients, but these were mostly negligible (differences in standardized coefficients within 0.01 to 0.03 for independent variables), except for the relationship between linear time and ES, where the effect size was decreased from *β* = 0.36 to *β* = 0.18. Item 9 was kept in the analyses to make the scale more similar when comparing scores between time points. The wording of the item was also deemed to indicate face validity.

#### 2.3.2. Mental Health

To measure mental health, the General Health Questionnaire (GHQ-12) [34] was used. GHQ-12 consists of a total of 12 items rated on a Likert scale ranging from 0 (strongly disagree) to 3 (strongly agree). Examples of items are “Have your worries made you lose a lot of sleep?” and “Have you felt unhappy and depressed”. Scoring on some items is flipped due to their wording. Higher scores indicate worse mental health. According to a meta-analysis, the GHQ-12 has shown moderate to high test-retest reliability [35]. The Swedish version has shown good internal consistency [36] and excellent discriminant validity [37]. In the present study, Cronbach’s alpha was measured at 0.75, 0.77, and 0.73 at T1, T2, and T3, respectively.

#### 2.3.3. Coach Interpersonal Style

To measure coach interpersonal style, a modified version of the Interpersonal Supportiveness Scale-Coach [38,39] was used. The scale originally measured coach interpersonal style on three subscales: autonomy, structure, and involvement [39]. However, Stenling et al. showed that autonomy, structure, and involvement could be brought together in a single subscale called need-supportive [40]. A subscale measuring controlling coach interpersonal style was added from a study of Smith et al. [41]. The scale consists of 21 items (nineteen for need-supportive and six for controlling) reported on a Likert scale ranging from 0 (not true at all) to 7 (very true), with higher scores indicating more use of that coach interpersonal style. Examples of items were “My coach cares about me” (need-supportive) and “My coach is excessively critical of me if I haven’t performed well in my sport” (controlling). The Swedish version of the questionnaire has previously shown acceptable internal consistency both regarding the need-supportive subscale [40] as well as the controlling subscale [42]. In the present study, Cronbach’s alpha was measured at 0.95, 0.97, and 0.96 for need-supportive and 0.74, 0.81, and 0.86 for controlling at T1, T2, and T3, respectively.

#### 2.3.4. Peer Motivational Climate

To measure peer motivational climate, the Peer Motivational Climate in Youth Sport Questionnaire (PeerMCYSQ) [24] was used. PeerMCYSQ consists in total of 21 items, with 12 items on task-involving peer motivational climate (with items such as “On my team, most athletes work together to improve the skills they don’t do as well”) and 9 items on ego-involving peer motivational climate (with items such as “On my team, most athletes criticize their teammates when they make mistakes”). All items were measured with a Likert scale ranging from 1 (strongly disagree) to 7 (strongly agree). Higher score indicates higher presence of that kind of peer motivational climate. Due to a need to limit the number of questionnaires, as reported by from the participants, the survey was too extensive, and no data on peer motivational climate was collected during the third time point. Therefore, the means of available time points will be used for ego-involving peer motivational climate and task-involving peer motivational climate, respectively. The factor structure of the Swedish version has previously shown an acceptable fit to the data [43]. In the present study, Cronbach’s alpha was measured at 0.93 and 0.93 for task-involving motivational climate and 0.78 and 0.82 for ego-involving motivational climate at T1 and T2, respectively.

### 2.4. Procedure for Data Collection in This Study

During T1 (first half of November 2013) and T2 (middle of April 2014), the participants filled out the questionnaires by pen and paper at school. During T3 (first half of November 2014), the questionnaires were answered digitally. This change in method was not considered an issue since previous research supports that digital administration of questionnaires leads to comparable results to pen-and-paper administration [44]. Furthermore, no clear pattern of higher or lower correlation between study variables comparing T1 to T2 (both pen and paper) and T2 to T3 (pen and paper and digital) was found (see Appendix A). Response rates also appeared equivalent across formats. Swedish versions were used for all questionnaires.

### 2.5. Data Analysis

All data analyses were conducted using Jamovi 2.3.28 (The jamovi project, Sydney, Australia) [45], except for Little’s MCAR test, which was conducted using SPSS v.28.0.1.1 (IBM, Armonk, New York, USA) [46]. Descriptive statistics for study variables were calculated in the form of means and standard deviations. ERQ is reported as item mean, while all other variables are reported as sums. Data was scrutinized for outliers. To investigate within-individual associations, linear mixed models [47] were conducted. Linear mixed models (LMM) are statistical methods that account for both fixed effects (consistent across all participants) and random effects (varying between participants), making them ideal for analyzing repeated measures data. The linear mixed models were estimated using restricted maximum likelihood to investigate how ES and CR relate to mental health, time effects on ES and CR, as well as how coach interpersonal style and peer motivational climate relate to ES and CR. Random intercept was set for subject in all models. Gender and type of sport (team sport or individual sport) were used as control variables in all models except for time effects on emotion regulation strategies. Gender was selected as a control variable due to adolescent females having more mental health problems [48], and since females tend to use more CR [49] and less ES [28] than males. Type of sport was selected as a control variable due to previous research indicating that athletes in individual sports are experiencing more mental health problems [50] and more use of ES [22] than athletes in team sports. For model simplicity, no interaction terms were included in the models. See Table 1 for a summary of models.

For the model investigating the effect of time on emotion regulation strategies, both linear and quadratic effects were studied in the case of the eventual changes, accelerating or decelerating over time. To facilitate interpretation of coefficients as standardized effect sizes, all variables, except for time point, were standardized using z-transformation. Satterthwaite’s formula was used to calculate degrees of freedom. Residuals were checked for normality and homoscedasticity. Random effects were checked for normality. Multicollinearity was considered by studying correlations between independent variables. Before Little’s MCAR test, all continuous variables were checked for normality using Shapiro–Wilks test. Variables not considered normally distributed were transformed using square root transformation. GHQ for time point 3 was the only variable not to reach normality after transformation (*W* = 0.97, *p* = 0.04). Little’s MCAR test [51] showed that data were missing completely at random, *χ*^2^ 512.24, *DF* = 504, *p* = 0.39. The level of significance was set at *p* < 0.05, and effect sizes were based on guidelines from Gignac and Szodorai [31], where effect sizes can be either small (*r* = 0.10), medium (*r* = 0.20), or large (*r* = 0.30).

## 3. Results

### 3.1. Descriptive Statistics

Means and standard deviations of study variables are presented in Table 2. The Pearson correlation matrix of study variables is available in Appendix A. None of the independent variables correlated above *r* = 0.70 with each other.

### 3.2. Emotion Regulation Strategies and Mental Health

Both ES, *β* = 0.34, *t* (208.21) = 5.07, *p* < 0.001, and CR, *β* = −0.33, *t* (213.43) = −5.06, *p* < 0.001, showed independent within-individual relationships to mental health problems with large effect sizes. See Table 3. These results indicate that when young elite athletes are using less ES, and more CR, they are also experiencing better mental health.

### 3.3. Changes in Emotion Regulation Strategies over Time

**Figure 1:**
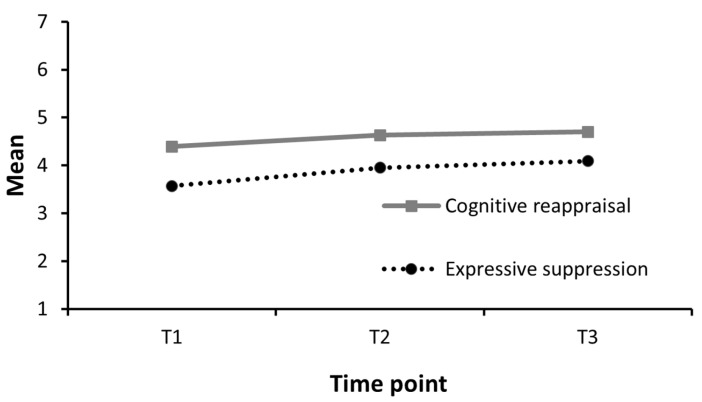
Changes in emotion regulation strategies over twelve months. Note: Changes in item means on Emotion Regulation Questionnaire subscales over the twelve-month study period.

Both ES, *β* = 0.36, *t*(154.19) = 4.37, *p* < 0.001, and CR, *β* = 0.23, *t*(152.49) = 2.65, *p* = 0.009, showed significant linear increase over the study period. Expressive suppression with a large effect size and CR with a medium effect size. This means that the young elite athletes were observed to report increased use of both ES and CR during this twelve-month period. The quadratic effects were nonsignificant for both ES, *β* = −0.07 *t*(154.88) = −0.82, *p* = 0.414, and CR, *β* = −0.06 *t*(152.37 = −0.72, *p* = 0.471, meaning that there were no differences in the paces of increase in the use of ES or CR between the first and second half of these twelve months. See Table 4 and Figure 1.

### 3.4. The Relationships Between Coach Interpersonal Style and Peer Motivational Climate and Emotion Regulation Strategies

Controlling coach interpersonal style, *β* = 0.20, *t*(210.77) = 2.88, *p* = 0.004, was significantly related to ES with a medium effect size, meaning that when the young elite athletes perceive their coaches as more controlling, they are also using more ES. Need-support coach interpersonal style (*β* = 0.19, *t*(204.88) = 2.72, *p* = 0.007), controlling coach interpersonal style (*β* = 0.20, *t*(203.47) = 2.92, *p* = 0.004), and task-involving peer motivational climate (*β* = 0.25, *t*(104.73) = 2.89, *p* = 0.005) were all significantly related to CR with medium effect sizes. This means that when the young elite athletes are perceiving their coaches as more controlling as well as more need-supportive, they are also using more CR. The young elite athletes who perceived the climate among their peers as more focused on mastery and saw errors as a part of the process were also using more CR. See Table 5.

## 4. Discussion

In line with the first hypothesis, more use of ES was related to worse mental health in young elite athletes, with an effect size (*β* = 0.34) that could be considered large according to Gignac and Szodorai [31]. This effect size suggests not only statistical significance but also practical significance, as it indicates that athletes relying heavily on ES experience meaningfully higher levels of mental health problems. This harmonizes with findings showing that habitual ES use is linked not only to acute distress but also to sustained mental health concerns over time in both college-aged athletes [12] and the general population [11]. The present study expands on those findings by showing that this relationship also exists within individuals, meaning that when someone uses more ES, they tend to experience worse mental health. This harmonizes with a meta-analysis of daily diary and experience sampling studies that found that ES was related to increased negative affect [52]. In addition, our findings demonstrate that this relationship also applies to habitual ES use over longer periods of time. Gross and John [31] suggest that ES is related to diminished ability to mood repair, meaning that the young elite athletes who rely on ES might be unable to mitigate the impact of increasing demands on their mental health. To summarize, when young elite athletes are making efforts to suppress their emotions, they are also experiencing worse mental health.

Also in line with the first hypothesis and previous research [11,12], more use of CR was related to better mental health in young elite athletes, with a large effect size [31]. Gross and John [32] suggest that the use of CR gives the opportunity to reinterpret stressful events with an optimistic attitude, and in that way, the young elite athletes might cope with increased demands in ways that are associated with better mental health. Potential interventions worth exploring in future studies that target CR could include cognitive-behavioral techniques training for coaches, reframing exercises, and perspective-taking training programs that help athletes reinterpret competitive stressors.

In line with the second hypothesis, the young elite athletes slightly increased their use of emotion regulation strategies during the twelve-month study period. This increase was regarding both ES and CR, meaning that they increased their use of both adaptive and maladaptive emotion regulation strategies. Given that previous research had not established clear trajectories for emotion regulation strategies during adolescence [13,14,15,16,17,18], these changes are likely to be influenced by environmental factors, rather than age. This increase in emotion regulation strategies could be explained by the young elite athletes aiming to cope with the increased demands during upper secondary sport education reported by Stambulova et al. [19]. These findings highlight the potential importance of the beginning of upper secondary education as a time period for promoting more adaptive emotion regulation strategies, such as CR.

For the third hypothesis, the study found mixed support. Both coach interpersonal style and the peer motivational climate were related to the emotion regulation strategies of the young elite athletes, although in different ways. Both the need-supportive coach interpersonal style and the controlling coach interpersonal style were related to increased use of CR, meaning that when coaches promote autonomy and structure and are involved in the well-being of the athlete, as well as when the coach takes a clear leading role and is punishing in cases of sport-related failures, the young elite athletes are more likely to reinterpret situations to regulate their emotions. This positive effect of the need-supportive coach interpersonal style is in accordance with, while the positive effect of the controlling coach interpersonal style is contrary to, previous research on basic psychological need satisfaction [22]. This seemingly paradoxical finding may be explained by the fact that both coaching styles present cognitive challenges that require reappraisal. Need-supportive coaching may encourage athletes to reframe challenges as growth opportunities, while controlling coaching may necessitate reappraisal as a coping mechanism to manage the stress of demanding expectations. The controlling coach interpersonal style was also related to increased use of ES, which is somewhat in contrast to Robazza et al., where the relationship between the sense of autonomy and ES was nonsignificant. However, Robazza et al. did not study controlling coach interpersonal style directly [22], which may explain the difference. This dual effect may arise from a heightened need for emotion regulation in response to a coach’s controlling interpersonal style, perhaps due to the young elite athlete being hesitant to express their initial emotion when unsure of the coach’s response.

Furthermore, a task-involving peer motivational climate was related to more use of CR, meaning that when peers promote mastery and individual improvement, the young elite athletes are more inclined to use CR. This harmonizes with previous research on the role of peer motivational climate on emotion regulation strategies [53] as well as the research on coach motivational climate [54]. However, contrary to previous research regarding coach motivational climate [54], in our study, which focuses on peer motivational climate, ego-involving motivational climate was unrelated to both CR and ES, and task-involving motivational climate was unrelated to ES. This could stem from the different impact that the coach and the peers might have on the young elite athletes [55,56].

### 4.1. Limitations and Further Directions

This study had a few limitations. First, all participants were students at the same school, limiting the generalizability of the results to other sports schools in Sweden and other countries. The participants were, however, heterogeneous in terms of sport types and gender distribution, suggesting that the school should be similar to other sports schools. Further research should replicate these findings using multiple schools to establish generalizability. Second, given the observational design, causality cannot be inferred. Future randomized controlled trials are needed to determine whether interventions targeting emotion regulation strategies can causally improve mental health in young elite athletes. A third limitation is the use of self-report measures of the behaviors of the coach and the peers. The perception of the behaviors of others may be influenced by the emotion regulation strategies of the athlete (e.g., an athlete using more CR might interpret the coach interpersonal style as more favorable). Future studies could address potential perception biases through triangulation methods, including coach self-reports, peer ratings, and objective behavioral observations to validate athlete perceptions. Fourth, even though both team and individual sports were represented in the study, the sample is somewhat skewed towards team sports (*n* = 78 for team sports, *n* = 14 for individual sports), limiting the generalizability of the results for athletes in individual sports. Fifth, regarding emotion regulation strategies, mental health, and coach interpersonal style, it was possible to study within-individual relationships. However, regarding peer motivational climate, it was only possible to study between-individual relationships since data from the third time point were lacking. Future studies should concurrently measure peer motivational climate, emotion regulation strategies, and mental health over time to establish within-individual relationships. Regarding reliability, ES did show poor internal consistency during T1. This was deemed to be caused by a single item. Reanalysis with this item removed was conducted and did not impact any relationships significantly, except for the increased use of ES during the study period, where the reanalysis still showed an increase, but the effect size was halved. This means that the finding of increased use of ES during the beginning of the young elite athletes’ upper secondary education should be interpreted with caution and would need replication to be properly established. Moreover, including multiple independent variables in the same model can cause problems regarding standard errors and *p*-values if these independent variables are too closely correlated to each other. However, in the present study, there was no correlation stronger than *r* = 0.70 between independent variables, and therefore, multicollinearity was not deemed to be a major issue.

### 4.2. Implications

The finding that both ES and CR were associated with mental health in young elite athletes suggests that facilitating CR over ES could be explored in future research as a possible strategy for supporting mental health in this population. Previous studies have demonstrated the malleability of emotion regulation strategies. In a randomized controlled trial, Samadi et al. showed an effect of a mindfulness-based intervention on both CR and ES in young athletes [57], and since use of ES and CR has been linked to a plethora of other outcomes in athletes, such as performance [58] and physical recovery [59], such interventions could be beneficial for athletes in multiple ways. Josefsson et al. also found a reduction in emotional dysregulation in athletes after a mindfulness-based intervention [60]. Moreover, the finding about the role of coach interpersonal style and peer motivational climate in the use of emotion regulation strategies could inform coaches and peers on how to act in ways that are associated with more adaptive emotion regulation strategies among athletes. For example, when a young elite athlete is lacking adaptive emotion regulation strategies, coaches should listen to the athlete’s ideas and give clear feedback, and the peers should be supportive when the athlete is trying their hardest. Since the first half of upper secondary education was found to be a period when the athletes increase the overall use of emotion regulation strategies, this is a favorable time for coaches and peers to facilitate the use of more effective forms of emotion regulation strategies. Further research could try to implement programs aiming to increase need-supportive coach interpersonal style and task-involving peer motivational climate to study the impact on emotion regulation strategies. See Table 6 for practical recommendations.

## 5. Conclusions

In young elite athletes, habitual use of CR was related to better mental health, while habitual use of ES was related to worse mental health. This was in line with the first hypothesis of the study, as well as previous studies, thereby contributing to existing literature by establishing this relationship in young elite athletes. Over the 12-month study period, the athletes demonstrated a slight increase in both ES and CR usage, indicating some dynamic shifts in their emotion regulation practices. This was somewhat in line with our second hypothesis that suggested that use of emotion regulation strategies would increase overall, but that it was unclear whether it would mainly be in the form of ES or CR. Furthermore, both coach interpersonal style and peer motivational climate were found to relate to the emotion regulation strategies employed by young elite athletes, although not completely in the hypothesized direction.

Overall, these findings highlight the potential for future randomized controlled trials where emotion regulation is modified in young elite athletes and their mental health is followed. Perhaps such interventions could be aimed at influencing coach behaviors or the culture between the peers to establish causality for those relationships as well. These studies should investigate the effectiveness and feasibility of such interventions.

In summary, this study provides the first longitudinal evidence that young elite athletes increasingly rely on both adaptive and maladaptive emotion regulation strategies over time, with ES being related to worse mental health and CR being related to better mental health. These findings could help to identify young elite athletes who are struggling with mental health issues.

## Figures and Tables

**Table 1:** Summary of models.

Model	Dependent Variable	Independent Variable	Purpose
1	Mental Health	ES, CR, Gender, Sport Type	Test H1: Emotion regulation-mental health associations.
2a	Expressive Suppression	Time (linear, quadratic)	Test H2: Temporal changes in expressive suppression.
2b	Cognitive Reappraisal	Time (linear, quadratic)	Test H2: Temporal changes in cognitive reappraisal.
3a	Expressive Suppression	Coach interpersonal styles, Peer motivational climate, Gender, Sport Type	Test H3: Predictors of expressive suppression.
3b	Cognitive Reappraisal	Coach interpersonal styles, Peer motivational climate, Gender, Sport Type	Test H3: Predictors of cognitive reappraisal

**Table 2:** Means and standard deviations of study variables.

Variable	T1 M(SD) N	T2 M(SD) N	T3 M(SD) N
Emotion regulation			
Cognitive reappraisal	4.39 (0.87) 78	4.63 (1.00) 82	4.70 (1.02) 73
Expressive suppression	3.57 (0.98) 78	3.95 (1.11) 82	4.09 (1.15) 78
Coach interpersonal style			
Need-supportive	106.76 (17.39) 75	107.39 (18.19) 80	109.82 (17.91) 77
Controlling	17.08 (6.48) 75	18.21 (7.48) 80	18.79 (8.03) 77
Peer motivational climate			
Task-involving	61.44 (12.71) 72	65.11 (11.20) 78	-
Ego-involving	34.15 (8.74) 75	34.42 (9.49) 81	-
Mental health	9.19 (4.24) 74	8.51 (4.48) 82	8.40 (3.01) 88

Note: Cronbach’s alpha values for all scales at each time point are provided in the Methods section. Abbreviations: M = Mean. N = Number of participants. SD = Standard deviation. T1 = First semester. T2 = Second semester. T3 = Third semester.

**Table 3:** Beta coefficients (and standard errors) for linear mixed models with mental health problems as dependent variable.

	Estimates
Intercept	0.20 (0.10)
ES	0.34 ** (0.07)
CR	−0.33 ** (0.07)
Gender †	0.44 ** (0.15)
Type of sport ††	0.47 * (0.21)
R-squared Marginal	0.19
R-squared Conditional	0.43
AIC	579.65
BIC	617.56
Random effect LRT	15.08 **

* *p* < 0.05 ** *p* < 0.01. † Male = 1, female = 2. †† Team sport = 1, individual sport = 2. Abbreviations: AIC = Akaike information criterion. BIC = Bayesian information criterion. CR = Cognitive reappraisal. ES = Expressive suppression. Random effect LRT = Random effect likelihood ratio test.

**Table 4:** Beta coefficients (and standard errors) for linear mixed models with ES and CR as dependent variables.

	ES	CR
Intercept	0.01 (0.09)	−0.01 (0.08)
Time point linear	0.36 ** (0.08)	0.23 ** (0.09)
Time point quadratic	−0.07 (0.08)	−0.06 (0.09)
R-squared Marginal	0.04	0.02
R-squared Conditional	0.49	0.44
AIC	631.10	629.10
BIC	657.89	655.57
Random effect LRT	43.29 **	36.33 **

** *p* < 0.01. Abbreviations: AIC = Akaike information criterion. BIC = Bayesian information criterion. CR = Cognitive reappraisal. ES = Expressive suppression. Random effect LRT = Random effect likelihood ratio test.

**Table 5:** Beta coefficients (and standard errors) for linear mixed models with ES and CR as dependent variables.

	ES	CR
Intercept	0.03 (0.13)	0.08 (0.11)
Need Support CIS	0.03 (0.07)	0.19 ** (0.07)
Controlling CIS	0.20 ** (0.07)	0.20 ** (0.07)
Task-involving PMC	−0.06 (0.09)	0.25 ** (0.09)
Ego-involving PMC	−0.05 (0.11)	−0.04 (0.10)
Gender †	−0.36 (0.20)	0.21 (0.18)
Type of sport ††	0.15 (0.26)	0.20 (0.23)
R-squared Marginal	0.07	0.20
R-squared Conditional	0.45	0.47
AIC	586.98	557.94
BIC	636.12	608.01
Random effect LRT	29.05 **	19.34 **

** *p* < 0.01. † Male = 1, female = 2. †† Team sport = 1, individual sport = 2. Abbreviations: AIC = Akaike information criterion. BIC = Bayesian information criterion. CIS = Coach interpersonal style. CR = Cognitive reappraisal. ES = Expressive suppression. PMC = Peer motivational climate. Random effect LRT = Random effect likelihood ratio test Abbreviations: AIC = Akaike information criterion. BIC = Bayesian information criterion. CR = Cognitive reappraisal. ES = Expressive suppression. Random effect LRT = Random effect likelihood ratio test.

**Table 6:** Practical recommendations for coaches and peers.

Target Group	Recommendation	Implementation Strategy
Coaches	Promote cognitive reappraisal.	Teach reframing techniques, model positive interpretation.
Coaches	Reduce controlling behaviors.	Focus on autonomy support, avoid excessive criticism.
Peers	Foster task-involving climate.	Emphasize effort over outcomes, normalize mistakes.
Peers	Provide emotional support.	Create safe spaces for emotional expression.

## Data Availability

The data that support the findings of this study are available on request from the corresponding author, F.F.R. Due to the inclusion of sensitive personal data, the data is not publicly accessible. Researchers interested in accessing the data for further analysis or replication of the study findings can reach out to the corresponding author for assistance.

## Abbreviations

The following abbreviations are used in this manuscript:

AIC	Akaike Information Criterion
BIC	Bayesian Information Criterion
CIS	Coach Interpersonal Style
CR	Cognitive Reappraisal
ERQ	Emotion Regulation Questionnaire
ES	Expressive Suppression
GHQ-12	General Health Questionnaire
LRT	Likelihood Ratio Test
M	Mean
N	Number of Participants
PeerMCYSQ	Peer Motivational Climate in Youth Sport Questionnaire
PMC	Peer Motivational Climate
SD	Standard Deviation

## Appendix A

**Table A1:** Pearson correlation matrix of study variables.

Variable	1	2	3	4	5	6	7	8	9	10	11	12	13	14	15	16	17	18	19
1. CR T1	-																		
2. CR T2	0.52 **	-																	
3. CR T3	0.42 **	0.40 **	-																
4. ES T1	0.03	−0.01	0.08	-															
5. ES T2	0.17	0.29 **	0.13	0.44 **	-														
6. ES T3	0.05	0.15	0.48 **	0.39 **	0.59 **	-													
7. GHQ T1	−0.18	−0.24 *	−0.19	0.35 **	0.24 *	0.10	-												
8. GHQ T2	−0.14	−0.31 **	0.03	0.16	0.25 *	0.21	0.62 **	-											
9. GHQ T3	0.07	−0.20	−0.05	0.01	0.01	0.13	0.11	0.30 **	-										
10. NS T1	0.29 *	0.29 *	0.08	0.01	0.12	0.08	−0.17	−0.19	−0.04	-									
11. NS T2	0.25 *	0.44 **	0.28 *	−0.03	0.06	0.17	−0.36 **	−0.29 **	−0.29 *	0.44 **									
12. NS T3	0.21	0.10	0.34 **	−0.02	0.04	0.09	−0.16	0.10	−0.12	0.29 *	0.61 **	-							
13. Cont T1	0.18	0.12	0.09	0.10	0.35 **	0.16	0.06	0.08	0.19	0.06	0.02	0.11	-						
14. Cont T2	0.09	0.04	−0.05	0.13	0.27 *	0.13	−0.03	0.07	0.16	0.05	−0.03	−0.01	0.63 **						
15. Cont T3	0.11	0.08	0.19	0.16	0.26 *	0.29 **	−0.20	0.08	0.15	0.02	0.02	−0.12	0.44 **	0.59 **	-				
16. Task T1	0.43 **	0.54 **	0.30 *	0.04	0.10	0.02	−0.38 **	−0.32 **	−0.10	0.57 **	0.48 **	0.27 *	0.06	−0.03	0.01	-			
17. Task T2	0.48 **	0.47 **	0.14	−0.19	−0.03	−0.06	−0.35 **	−0.31 **	−0.13	0.47 **	0.55 **	0.33 **	0.10	0.09	−0.03	0.65 **			
18. Ego T1	−0.14	−0.14	−0.14	0.05	0.16	0.02	0.12	0.01	−0.15	0.06	−0.12	−0.00	0.33 **	0.42 **	0.25 *	−0.29 *	−0.25 *		
19. Ego T2	−0.24 *	−0.12	−0.04	0.12	0.16	0.17	0.04	−0.08	−0.03	−0.04	−0.12	−0.04	0.29 *	0.55 **	0.34 **	−0.16	−0.21	0.67 **	-

* *p* < 0.05 ** *p* < 0.01. Abbreviations: CR = Cognitive reappraisal. ES = Expressive suppression. GHQ = General health questionnaire. NS = Need-support coach interpersonal style. Cont = Controlling coach interpersonal style. Task = Task-involving motivational climate. Ego = Ego-involving motivational climate. T1 = First semester. T2 = Second semester. T3 = Third semester.

## Appendix B

### Analysis of Internal Consistency for ES at T1

Cronbach’s alpha for ES at T1 was 0.44. Table A2 shows means, standard deviations, item-rest correlations, and Cronbach’s alpha if item is dropped. Table A3 shows between-item correlations.

**Table A2:** Descriptive statistics for expressive suppression at time 1.

	Mean	SD	Item-Rest Correlation	Cronbach’s Alpha If Item Dropped
Item 2	4.36	1.56	0.24	0.38
Item 4	3.42	1.88	0.46	0.09
Item 6	3.69	1.61	0.49	0.10
Item 9	2.81	1.35	−0.14	0.65

**Table A3:** Between-item correlations.

	1	2	3
1. Item 2	-		
2. Item 4	0.19	-	
3. Item 6	0.54	0.44	-
4. Item 9	−0.34	0.18	−0.20

Since item 9 correlated poorly to the other items, a reanalysis was made with item 9 removed, i.e., a new measure of ES was constructed consisting of items 2, 4, and 6. The new measure had a Cronbach’s alpha of 0.65.

Confirmatory factor analysis with items 1, 3, 5, 7, 8, and 10 loading on the factor cognitive reappraisal and items 2, 4, 6, and 9 loading on the factor expressive suppression was conducted (model 1). Standardized estimates showed that item 9 loaded negatively (−0.22) on the factor expressive suppression. A second model (model 2), removing item 9, showed a comparable better fit to the data as indicated by *χ^2^*/*df*, CFI, and RMSEA (Table A4).

**Table A4:** Model fit for the model, including item 9 (model 1) and the model with item 9 removed (model 2).

	*χ* ^2^	*df*	*χ*^2^/*df*	CFI	RMSEA
Model 1	65.96 **	34	1.94	0.85	0.11
Model 2	46.44 **	26	1.79	0.90	0.10

** *p* < 0.01.

All linear mixed models, including ES as a variable, were re-run with this new measure of ES, where item 9 is removed. Table A5 shows the main analysis conducted with this new measure, Table A6 shows the time point as the independent variable, and Table A7 shows peer motivational climate and coach interpersonal style as independent variables. Estimates from the original measurements are included for clarity.

**Table A5:** Beta coefficients (and standard errors) for linear mixed models with mental health problems as dependent variables.

	Estimates with New ES Measure	Estimates with Original ES Measure
Intercept	0.19 (0.14)	0.20 (0.10)
ES	0.35 ** (0.07)	0.34 ** (0.07)
CR	−0.33 ** (0.07)	−0.33 ** (0.07)
Gender ^†^	0.43 ** (0.16)	0.44 ** (0.15)
Type of sport ^††^	0.45 * (0.21)	0.47 * (0.21)
R-squared Marginal	0.20	0.19
R-squared Conditional	0.46	0.43
AIC	577.62	579.65
BIC	615.43	617.56
Random effect LRT	18.47 **	15.08 **

* *p* < 0.05 ** *p* < 0.01. † Male = 1, female = 2. †† Team sport = 1, individual sport = 2. Abbreviations: AIC = Akaike information criterion. BIC = Bayesian information criterion. CR = Cognitive reappraisal. ES = Expressive suppression. Random effect LRT = Random effect likelihood ratio test.

**Table A6:** Beta coefficients (and standard errors) for linear mixed models with expressive suppression as dependent variable.

	**Estimates with New ES Measure**	**Estimates with Original ES Measure**
Intercept	0.00 (0.09)	0.01 (0.09)
Time point linear	0.18 * (0.08)	0.36 ** (0.08)
Time point quadratic	0.03 (0.08)	−0.07 (0.08)
R-squared Marginal	0.01	0.04
R-squared Conditional	0.49	0.49
AIC	635.37	631.10
BIC	662.11	657.89
Random effect LRT	46.47 **	43.29 **

* *p* < 0.05 ** *p* < 0.01. Abbreviations: AIC = Akaike information criterion. BIC = Bayesian information criterion. CR = Cognitive reappraisal. ES = Expressive suppression. Random effect LRT = Random effect likelihood ratio test.

**Table A7:** Beta coefficients (and standard errors) for linear mixed models with expressive suppression as dependent variable.

	**Estimates with New ES Measure**	**Estimates with Original ES Measure**
Intercept	0.03 (0.13)	0.03 (0.13)
Need Support CIS	0.04 (0.07)	0.03 (0.07)
Controlling CIS	0.17 * (0.07)	0.20 ** (0.07)
Task-involving PMC	−0.07 (0.10)	−0.06 (0.09)
Ego-involving PMC	−0.05 (0.11)	−0.05 (0.11)
Gender ^†^	−0.33 (0.21)	−0.36 (0.20)
Type of sport ^††^	0.16 (0.27)	0.15 (0.26)
R-squared Marginal	0.05	0.07
R-squared Conditional	0.52	0.45
AIC	578.92	586.98
BIC	627.70	636.12
Random effect LRT	41.14 **	29.05 **

* *p* < 0.05 ** *p* < 0.01. † Male = 1, female = 2. †† Team sport = 1, individual sport = 2. Abbreviations: AIC = Akaike information criterion. BIC = Bayesian information criterion. CIS = Coach interpersonal style. CR = Cognitive reappraisal. ES = Expressive suppression. PMC = Peer motivational climate. Random effect LRT = Random effect likelihood ratio test.

In summary, compared with the analyses using all four items for ES at time 1, all significant effects remained, and no new significant effects emerged. The differences in standardized coefficients were mostly negligible, spanning between 0.01 and 0.03 for the independent variables, except for the effect of linear time on ES, where the effect decreased from *β* = 0.36 to *β* = 0.18.
